# Sensory and Olfactometry Chemometrics as Valuable Tools for Assessing Hops’ Aroma Impact on Dry-Hopped Beers: A Study with Wild Portuguese Genotypes

**DOI:** 10.3390/foods10061397

**Published:** 2021-06-17

**Authors:** Júlio C. Machado, Florian Lehnhardt, Zita E. Martins, Miguel A. Faria, Hubert Kollmannsberger, Martina Gastl, Thomas Becker, Isabel M. P. L. V. O. Ferreira

**Affiliations:** 1 LAQV/REQUIMTE, Laboratório de Bromatologia e Hidrologia, Departamento de Ciências Químicas, Faculdade de Farmácia, Universidade do Porto, Rua de Jorge Viterbo Ferreira no. 228, 4050-313 Porto, Portugal; jcmjunior@ff.up.pt (J.C.M.J.); zmartins@ff.up.pt (Z.E.M.); mfaria@ff.up.pt (M.A.F.); 2 Chair of Brewing and Beverage Technology, Technische Universität München, Weihenstephaner Steig 20, 85354 Freising, Germany; florian.lehnhardt@tum.de (F.L.); h.kollmannsberger@mytum.de (H.K.); martina.gastl@tum.de (M.G.); tb@tum.de (T.B.)

**Keywords:** wild hops, olfactometry, aroma compounds, beer, CATA, quantitative descriptive analysis, certified assessors, multivariate statistical analysis

## Abstract

Sensory, olfactometry (using the sums of odour intensities for each class of compounds) and chemometric analyses were used to evaluate Portuguese wild hops’ sensory characteristics and the aroma that those hops impart to dry-hopped beer. CATA analysis and agglomerative hierarchical clustering was applied for the sensory characterization of 15 wild hops of Portuguese genotypes, clustering them in two groups: one more sulphurous, floral, and fruity, and another more earthy, resinous, floral, and non-citrus fruits. Two hops representative of each group were selected for the production of four dry-hopped beers using the same base beer style (Munich Helles). Beers were analysed by quantitative descriptive analyses and quantification of hop-derived key volatile compounds. Multivariate statistical treatment of the data was performed. Results indicate significant differences (*p* < 0.05) in fruity, resinous, earthy, floral, and sulphurous attributes of hops, but the dry-hopped beers only have a significant increase (*p* < 0.05) in fruity and spicy notes when compared with non-dry-hopped Munich-style Helles beer. Hop olfactometry explained the sensory perception that the 11 hops not used for brewing (employed as supplementary observations) are placed into the space of the odour-active compounds profile of the four hops selected for brewing. These 11 hop samples have more spiciness than fruitiness potential.

## 1. Introduction

Hop (*Humulus lupulus* L.) is the ingredient that adds bitterness, microbial protection, foam stability and flavour to beer [1]. Hop inflorescences of the female plant, referred to as cones, contain lupulin, which is formed by non-volatile resins and volatile essential oils, including a variety of hydrocarbons, sulphur and oxygenated compounds [2]. The addition of hops at the cold stages of brewing production, called dry-hopping, is a worldwide trend that is becoming increasingly popular for imparting diverse and intense hoppy flavour [3,4].

Consequently, there is a growing search for annual releases of new varieties of hops by hop growers and suppliers, following worldwide trends for flavour diversity in beer. Special attention is given to wild, unexplored plants and to their potential application in breeding programs, which means diverse genetic and environmental effects on hops composition and, consequently, a great number of hops to be analysed for their brewing properties. In Portugal, several wild hop populations were identified, revealing a large variability in morphologic [5], volatile profile and sensory characterization [6]. However, no published studies were found concerning the impact of Portuguese wild hops on the aromatic characteristics of beer.

Sensory evaluation is the most common tool for characterizing hops and presuming their influence on beer flavours. Sensory analysis of different hops reveals a wide variety of specific odour attributes, such as fruity (citrus, green, sweet, berries, and tropical fruits), vegetal (resinous/woody, floral, and herbal), spicy, and sulphurous notes [7,8]. However, the sensory perception of beer results from a high number of factors and predicting beer flavours is very complex due to synergistic, antagonistic, and masking chemical effects that occur [3,4].

Some studies dealing with the prediction of the sensory perception of beers take into consideration not only the sensory profile, but also the content of volatile compounds in hops [9,10,11,12,13,14]. However, to the best of our knowledge, no reports have used olfactometry for that purpose, although the methodology has already been useful for identifying and characterizing hops-derived compounds in beers [15,16,17].

The goal of this study was to use an innovative approach that combines hops olfactometry (using the sums of odour intensities for each class of compounds) with beer sensory and chemometric analyses to explain the aromatic perception that hops can impart to beer by dry-hopping. These tools were applied in a factual scenario for the evaluation of the aromatic impact of Portuguese wild hops on Munich-style Helles beer, after the analysis of a reduced and representative number of samples.

## 2. Materials and Methods

### 2.1. Chemical Reagents

Reference standards (+) -β-Pinene (≥98.5%), 1-Octen-3-ol, borneol (≥95%), butyric acid (≥99.5%), citral (≥95%), cis-3-hexen-1-ol (≥98%), diacetyl, dimethyl sulphide, dimethyl trisulphide (≥98.5%), 3-methylbutanal (≥97%), 2-methylbutanal (≥95%), ethyl 2-methylbutanoate (99%), ethyl 2-methylpentanoate (internal standard, ≥99%), ethyl-3-methylbutanoate (≥98%), 3-methylbutyl-2-methylpropanoate (≥98%), 2-methylbutyl-2-methylpropanoate (≥95%), and ethyl cinnamate (99%), ethyl 2-methylpropanoate (≥99%), ethyl 3-methylbutanoate (≥98%), ethyl 4-methylpentanoate (≥97%), ethyl butanoate (≥99,5%), ethyl hexanoate (≥99.5%), eugenol (99.6%), furaneol (≥99%), geraniol (≥99%), hexanol (≥99%), 3-methylbutyl acetate (≥97%), linalool (97%), menthol (99%), methyl nonanoate (≥99.8%), myrcene (≥90%), geranyl acetate (≥97%), dimethyl disulphide (≥90%), limonene (97%), hexanal (≥98%), 3-hexenol (≥95%), 2-phenyl ethanol (≥99%), β-caryophyllene (≥80%), α-humulene (≥96%), humulene oxide, S-methyl 5-methylpentanthioate S-methyl hexanthioate, S-methyl 4-methylpentanoate and theaspirane (≥90%) were purchased from Sigma Aldrich (St. Louis, MO, USA).

### 2.2. Hops Material

Inflorescences of 15 Portuguese wild hops collected in the natural environment (coded as PT1 to PT15) were dried at 60 °C for 6 to 8 h (moisture less than 8%), closed in vacuum bags, and stored in the absence of light at 4 °C until the moment of analysis. Reference varieties Ariana (ANA), Bravo™ (BRO), Hallertauer Blanc (HBC), Hallertauer Magnum (HMG), Hallertauer Taurus (HTU), Hallertauer Tradition (HTR), Herkules (HKS), Hersbrucker (HEB), Hüll Melon (HMN), Mandarina Bavaria (MBA), Opal (OPL), Polaris (PLA), Smaragd (SGD)—all harvested in 2018—were supplied by the Gesellschaft fuer Hopfenforschung e.V. (Wolnzach, Germany).

### 2.3. Dry-Hopping Trials

Four Portuguese hop samples were selected for dry hopping experiments in commercial Munich-style Helles beers (Freising, Germany) presenting original gravity: 11.6 °P, alcohol (ABV): 5.1% EBC and bitterness: 21 IBU. Dry-hopped beers were prepared in 10 L kegs (Cornelius Deutschland GmbH, Langenfeld, Germany). After hops addition (3 g/L), kegs were closed and filled with CO_2_ (1.5 bar) to carry out the procedure free of oxygen. Beer (8 L) was added and kegs were kept in agitation for 6 days at 4 °C. A similar procedure was carried out on a control (not dry-hopped beer), but without the addition of hops. All trials were done in triplicate. Hop concentration and maturation time were determined, taking into consideration previous studies of transfer rates of volatile compounds in dry-hopping techniques and the practical uses of breweries [17,18,19].

### 2.4. Sensory Evaluation

Sensory analyses were conducted on 15 hop samples and 4 dry-hopped beers. Assays were performed individually in standard cabins, and samples were presented blind-labelled with a three-digit code in triplicate at room temperature (20 ± 1 °C).

Hops were presented in original stored vacuum bags. The Check-All-That-Apply test (CATA) with a semi-trained panel of 25 individuals was performed to evaluate the hops’ odour [6]. In the training sessions, standard references and known commercial varieties of hops (Table 1) were presented to panellists to define hop attributes. After training sessions, the participants were able to detect differences between citrus and not citrus fruits, resinous, earthy, floral, green grassy, green tea, spicy, and sulphurous attributes in the selected hop samples. Earthy, green tea and sulphurous were described as undesired or unpleasant attributes of hops.

Beers analyses were performed on fresh samples (after 6 days of maturation time). The flavour profile of beer samples was conducted by a quantitative descriptive analysis approach performed by 10 assessors (20–50 years of age) certified by the German agricultural society (Deutsche Landwirtschafts-Gesellschaft e.V.) for the evaluation of beer sensory characteristics. Total hoppy impression, and the same attributes selected for hops, were analysed in beers. Each assessor rated the descriptors’ intensity on a five-point scale (0 = imperceptible, 1 = very weak, 2 = weak, 3 = middle, 4 = intensive and 5 = very intensive); the averaged results for each beer were plotted in a radar diagram. Preferences were analysed by liking score; panellists rated the beers on a four-point scale (0 = very bad, 1 = bad, 2 = good and 3 = very good).

### 2.5. Gas-Chromatography for Quantification of Beer Volatile Compounds and Olfactometry of Hops

Volatile compounds were extracted by way of headspace solid-phase micro-extraction. Beers (5 g) and hops (0.5 g) samples were placed in 20 mL headspace vials with polypropylene caps (Butyl/ PTFE, Achroma, Mühlheim, Germany) exposed to a divinylbenzene/carboxen/polydimethylsiloxane (DVB/CAR/PDMS) SPME fibre 50/30 μm (Supelco/Sigma Aldrich, Bellafonte, PA, USA) for 30 min at 40 °C and were analysed with gas chromatography-mass spectrometry-olfactometry (GC-MS/O) [17]. 

Chromatographic analysis was performed in the gas chromatograph system TRACE 1300 Ultra, directly coupled with an ISQ QD single quadrupole mass spectrometer (ThermoScientific, Waltham, MA, USA) equipped with an injection split/splitless port associated with a selective detector mass, EI mode at ionization energy of 70 eV. The GC-MS was equipped with a Trace GOLD TG-5MS (ThermoScientific Waltham, MA, USA) column (60 m × 0.25 mm × 0.25 mm). After volatiles extraction, HS-SPME fibre was desorbed (manually) in the injection port at 250 °C for 0.5 min in splitless mode. For the chromatographic separation, the GC oven temperature started at 60 °C, was held for 4 min, increased at 5 °C per minute to 220 °C, held for 5 min and heated to 250 °C at a rate of 10 °C per minute and the final temperature was held for 2 min. The transfer line was set to a temperature of 250 °C. The mass spectrometer detected mass ranges between 35 and 350 °u. The chromatographic separation had a constant flow of 1.2 mL/min using helium BIP as a carrier gas. The retention index (RI) of each compound was calculated using the retention time (RT) of that compound compared against the RTs of a series of standard n-alkanes. The compounds were identified based on their retention indices, odour perceptions, mass spectra of NIST 11 library, and authentic standards measured under the same conditions.

The quantification of volatile compounds in beer samples was conducted using an internal standard (IS, ethyl 2-methylpentanoate 0.02 µg/mL). Seven esters (ethyl 2-methylpropanoate, 2-methylbutyl 2-methylpropanoate, 3-methylbutyl 2-methylpropanoate, ethyl 2-methylbutanoate, ethyl 3-methylbutanoate, ethyl 4-methylpentanoate, and ethyl cinnamate), two monoterpenes (myrcene and geranyl acetate), two monoterpenoid alcohols (linalool and geraniol), two sesquiterpenes (β-caryophyllene and α-humulene), one sesquiterpenoid oxide (humulene oxide), one thiol/ sulphide (dimethyl trisulphide—DMTS), and two thioesters (S-methyl 5-methylpentanthioate and S-methyl hexanthioate) were selected as relevant flavouring beer compounds. Calibration curves at six concentration points were made on the beer matrix (Munich-style Helles).

Odour-active volatile compounds of hops were identified by olfactometry in the same gas chromatographic system. A trained GC-O analyst was asked to describe the perceived odours as well as their intensity. The method of odour intensity was used with a 4-point scale (not detected, weak, moderate, and strong). For each hop, the odour-active compounds were identified and grouped into classes according to their chemical functional group. The sums of odour intensities for each class of compounds were calculated.

### 2.6. Statistical Analysis

Cochran’s Q test [20] was performed with data obtained from CATA tests applied to hop samples, to determine whether attributes differed as a function of the hop sample. If there was a significant difference among the variables, post hoc multiple pairwise comparisons were performed using the critical difference (Sheskin) procedure. The sum of significant attributes across assessors was used to construct (i) a dendrogram by agglomerative hierarchical clustering (using Ward’s method of agglomeration and Euclidean distance dissimilarity) for studying the proximity between the hop samples; (ii) a correspondence analysis (using the Chi-square distance to test the independence between the hops and attributes) to verify how the hops and attributes were relatively positioned.

Regarding beer flavour profiles, a comparison of sensory quantitative descriptive analysis scores between non-dry-hopped and dry-hopped beers was carried out by t-test for two independent samples, or by Mann-Whitney two-tailed tests depending on whether a normal distribution of the residuals (using Shapiro–Wilk’s test) was confirmed or not, respectively. Data from the quantification of beer volatile compounds were analysed by multiple pairwise comparisons using the Conover-Iman procedure/two-tailed test since a normal distribution of residues (using Shapiro–Wilk’s test) was not confirmed. 

Principal component analysis (PCA) was performed using sensory and volatiles data from dry-hoped beers as active variables and liking scores as a supplementary variable (passive). Supplementary variables have no influence on the component factors’ definition, but plotting their categories on the factor plane can enrich the interpretation of the PCA output if some pattern emerges. Another PCA was performed using the olfactometry data of four selected hops as active variables, while sensory and volatiles data of dry-hopped beers were inserted as supplementary variables, and the olfactometry data of the remaining 11 hops as observational variables. In both PCA analyses, Pearson correlations were used to test the association between the active and supplementary variables. Pearson correlation coefficients indicate strong negative (r ≤ −0.80) and positive (r ≥ 0.80) correlations.

All statistical analyses were performed at a 5% significance level, using XLSTAT^®^ for Windows trial versions 2021.1.1.1092 (Addinsoft, Paris, France). 

## 3. Results

### 3.1. Sensory Analysis of Portuguese Wild Hops

Data from the CATA analysis was evaluated for the sensory characterization of the 15 Portuguese wild hops (Table 2). Resinous was the attribute perceived by most panellists. In almost all samples, except for PT01, the attribute was checked by at least half of the judges, being identified by at least 75% of assessors in PT03, PT05, PT06, PT07, PT09, PT10 and PT13. Regarding other attributes, it can be emphasized that 85% of assessors noted citrus fruits in PT14; non-citrus fruits were identified by 90% of the judges in PT14 and 75% in PT11. Moreover, 80% of panellists checked the floral characteristic for PT14 and spicy notes in PT05. Cochran’s Q test determined that the attributes citrus and non-citrus fruits, resinous, earthy, floral and sulphurous differed significantly as a function of hop samples (Table 2). Regarding pleasant attributes, the highest number of checks was observed in PT14 for citrus, and in PT11, PT14, and PT15 for non-citrus fruits. Resinous was mostly perceived in PT05 and PT07 and floral in PT14. On the other hand, panellists detected unpleasant earthy and sulphurous attributes mostly in PT07 and PT08, respectively.

**Figure 1:**
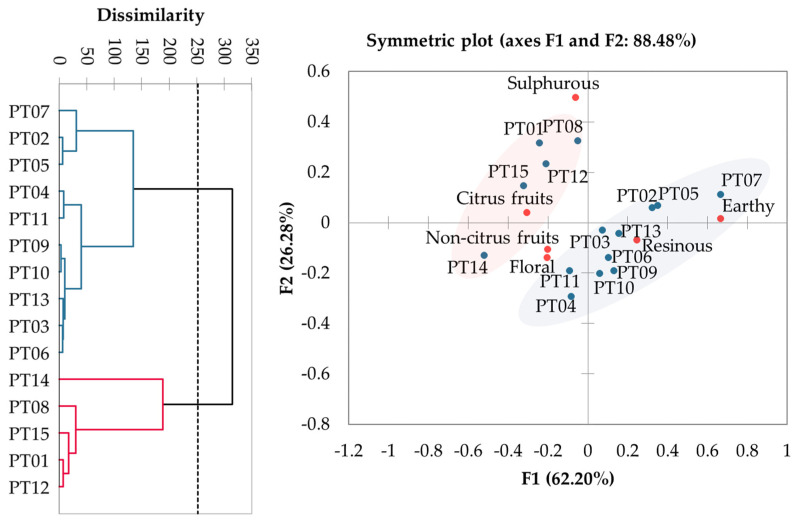
Agglomerative Hierarchical Clustering and Correspondence Analysis of Portuguese wild hops sensory attributes.

The independence between the sum of attribute tables across panellists (Supplementary Table S1) was tested using the Chi-square distance, showing real differences between the hops in terms of their sensory profiles (*p* = 0.02). A dendrogram was constructed by Agglomerative Hierarchical Clustering, grouping the hops into two clusters by similarity (Figure 1). Group 1 (G1) included PT01, PT08, PT12, PT14 and PT15, and group 2 (G2) presented PT02-07, PT09-11 and PT13 hops. A Correspondence Analysis was plotted to visualize how the hops and attributes are relatively positioned. With 88.48% of explained total variance on the first two dimensions, it is possible to observe that samples of G1 were classified as sulphurous—mainly PT01, PT08 and PT12—also floral and both fruity attributes were predominant for PT14 and PT15. Hops included in G2 are more earthy (mostly PT07), resinous (mainly PT02, PT03, PT05, PT06, PT09, PT10 and PT13), as well as floral and non-citrus fruits, chiefly PT04 and PT11.

After preliminary evaluation of the sensory attributes of the 15 studied Portuguese hops, two samples representative of each group were selected for beer production, taking into consideration the results obtained. PT05 and PT11 of G1, and PT14 and PT15 from G2 were selected since they had more balance with the pleasant fruity, floral, and resinous attributes, and were not related to unpleasant earthy or sulphurous sensations. 

### 3.2. Sensory Analysis of Beers Dry-Hopped with Selected Samples

**Figure 2:**
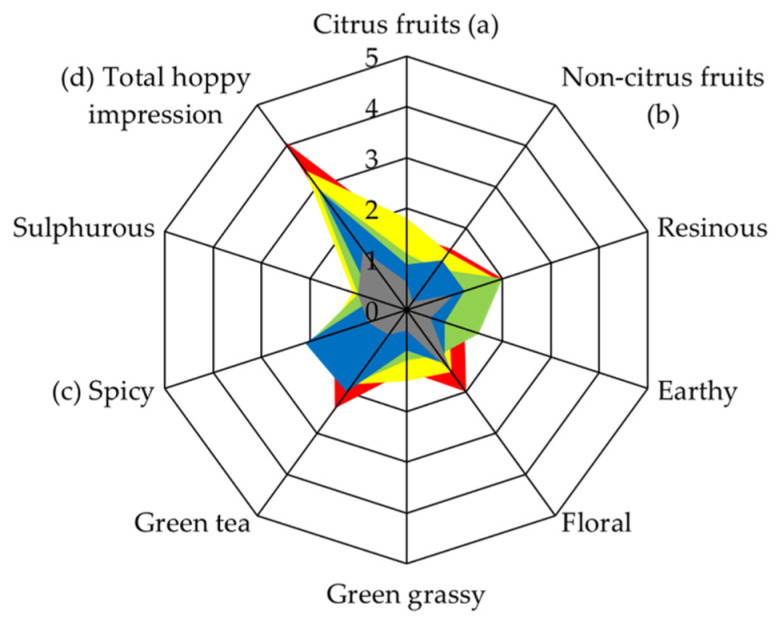
Quantitative descriptive sensory analysis of beers dry-hopped with Portuguese wild hops. Each beer score is represented by a different colour. PT05 (blue), PT11 (red), PT14 (yellow), PT15 (green), and non-dry-hopped beer in the grey area. Data from sensory analysis performed by trained panellists are presented as means (floral) in normal distribution or medians (other attributes) in non-normal distribution. Two independent samples t-test and Mann-Whitney test were applied to detect which attributes were significant in normal and non-normal distributions. Letters between brackets represent that (**a**) PT14, (**b**) PT11, (**c**) PT05, and (**d**) all hops presented significant statistical differences (*p* < 0.05) of the attribute in comparison with non-dry-hopped beer.

Dry-hopped beers were prepared using the four selected hops to verify how sensory attributes are transferred to beers. The base beer (non-dry-hopped) was used for comparison. Data from the quantitative descriptive analysis of base and dry-hopped beers are presented in Figure 2, this provides the global sensation perceived (olfactory and gustatory) when the product is evaluated. Beers dry-hopped with PT05, PT11 and PT14 hops presented a significant increase in spicy, non-citrus fruits, and citrus perception, respectively. Although, in general, these results match with CATA sensory analyses of these hop cones (Table 2), the association between the sensory perceptions of hops and the respective dry-hopped beers is not always clear. Hops from the same sensory clusters would be expected to produce dry-hopped beers with similar sensory perceptions, but this was not observed. Dry-hopped beers from PT11 (G1) and PT14 (G2) hops presented similar hoppy and fruity intensities. Additionally, some hops attributes, such as floral in PT14 and non-citrus fruits in PT14 and PT15, were noted in hops but not in the respective dry-hopped beers.

In a recent study, authors calculated the probability of perceiving a particular hop aroma in beer given by perceiving the aroma in hops. High probabilities were demonstrated for spicy, resinous, herbal, and grassy (100%), but only medium probabilities for citrusy (40%), floral (38%) and fruity (43%) aromas [21]. This is in general accordance with our results, where some positive associations, as well as some divergences, were demonstrated between the sensory analysis of hops and respective dry-hoped beers, mainly for floral and non-citrus fruit sensations. Therefore, although sensory analysis of hops can be useful for predicting some attributes of dry-hopped beers, it cannot be used to comprehensively anticipate their final sensory attributes. Combinatory, competing, masking, antagonistic, and synergistic chemical effects may occur and be the reason for some odours to be present in hops but not in beer, as well as the opposite [3,4]. Moreover, different aroma compounds responsible for the specific sensory attributes are expected to have different extraction patterns. The quantification of individual volatile compounds is important to have a better understanding and interpretation of sensory analysis results. In that sense, the aroma composition of dry-hopped beers and hops was evaluated to understand the aromatic characteristics that Portuguese wild hops impart to dry-hopped beer.

### 3.3. Aroma Composition of Beers Dry-Hopped with Selected Samples

Twelve key aroma compounds from hops that would be expected to be transferred to beer during the dry-hopping process were selected and quantified in beers. Esters, terpenes and sulphurous compounds have been cited by various authors as key hops compounds relevant to flavouring beers by dry-hopping techniques [18,22,23,24]. Therefore, seven esters (ethyl 2-methylpropanoate, 2-methylbutyl 2-methylpropanoate (2MB2MP), 3-methylbutyl 2-methylpropanoate (3MB2MP), ethyl 2-methylbutanoate, ethyl 3-methylbutanoate, ethyl 4-methylpentanoate, and ethyl cinnamate), two monoterpenes (myrcene and geranyl acetate), two monoterpenoid alcohols (linalool and geraniol), two sesquiterpenes (β-caryophyllene and α-humulene), one sesquiterpenoid oxide (humulene oxide), one thiol (dimethyl trisulphide (DMTS)), and two thioesters (S-methyl 5-methylpentanthioate and S-methyl hexanthioate), making a total of 12 compounds, were quantified in the four dry-hopped beers.

Concentrations and thresholds (µg/L) of volatile compounds in non-dry-hopped beer and beers dry-hopped with Portuguese wild hops are summarized in Table 3. Overall, the results obtained after compounds quantification agree with the sensory analysis. Significant differences (*p* < 0.05) were observed in the volatile compounds content of dry-hopped beers when compared with non-dry-hopped beer, which can explain the increased total hoppy impression in the sensory analysis. Regarding the monoterpenes, PT05 and PT15 hops promoted concentrations over the threshold levels for myrcene and the monoterpenoid alcohols linalool and geraniol (only PT05), but PT11 and PT14 were the hops that imparted the highest concentrations. Significant increases of monoterpene myrcene (in both beers) and the monoterpenoid alcohols linalool (in both) and geraniol (in PT11 dry-hopped beer) were observed. These compounds have been described with positive correlation to the intensity of hoppy aroma in dry-hopped beers [11,25]. 

In addition, PT11 and PT14 beers were the only ones that presented significant increases with values over the threshold of esters. The results obtained can explain the fruitiest sensation of these beers (Figure 2), since this class of compounds has been associated with hop-derived fruity characteristics of beers [11,26,27]. Therefore, PT11 presented the highest contents of ethyl 2-methylbutanoate, ethyl 3-methylbutanoate and ethyl 4-methylpentanoate, and PT14 of ethyl 2-methylpropanoate, 3-methylbutyl 2-methylpropanoate, 2-methylbutyl 2-methylpropanoate and ethyl cinnamate.

Sesquiterpenes are a class of compounds related to the resinous sensorial characteristics of hops, and their oxidation products (sesquiterpene oxides) are associated with the spicy sensation in beers [28,29,30]. From CATA analysis, PT05 and PT11 were the hops that presented the highest resinous intensity and, consequently, significant spicy notes in PT05 dry-hopped beer. Accordingly, PT05 and PT11 were the hops that promoted significant differences in the concentration of humulene oxide, even though the values were below the detection limits. β-caryophyllene and α-humulene were highest in PT14 dry-hopped beer. 

Neither the selected hops nor their dry-hopped beers presented increased sulphurous sensory perception. However, thiols were quantified, taking into consideration that they can add pleasant aromas to food and beer depending on molecular-weight and concentration [31,32]. A significant increase was found in the composition of the three tested compounds. All dry-hopped beers presented DMTS and S-methyl thiohexanoate over the minimum threshold levels. PT11 promoted the highest levels of DMTS and S-methyl 5-methylpentanthioate, and PT14 of S-methyl thiohexanoate. It is possible that these compounds influence the odour of dry-hopped beers, since fruity sensations are expected in residual concentrations for small and medium chains of S-methyl thioesters [33]. 

**Figure 3:**
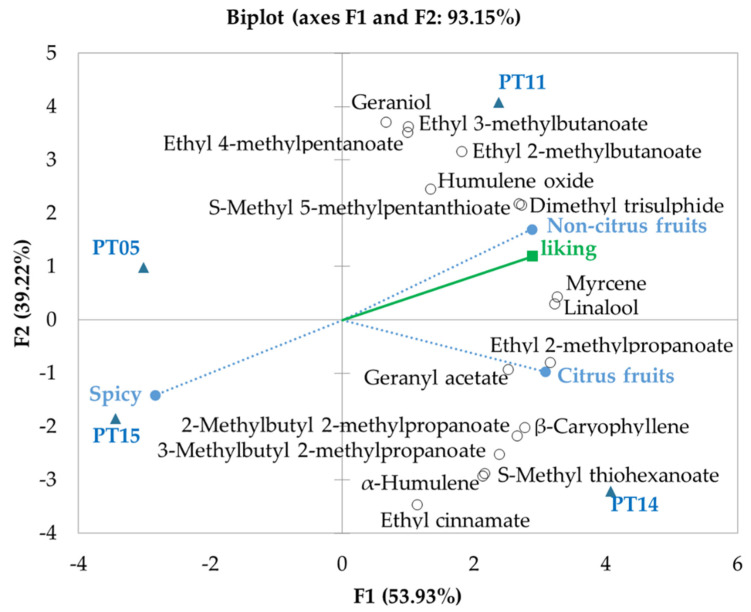
Principal Component Analysis (PCA) biplots of sensory attributes (blue circle) and volatile compounds content (black open circles) of dry-hopped beers (blue triangles) used as active variables. Liking scores (green square) were used as a passive (supplementary) variable.

Panellists also scored the beers according to preference on a four-point scale, where 0 means very bad, 1 is bad, 2 is good, and 3 is a very good beer. On average, PT05 (2.0 ± 0.6), PT11 (2.1 ± 0.9), and PT14 (2.1 ± 0.6) were classified as good, and PT15 0.9 ± 0.8 as a hedonically unpleasant beer. Principal Component Analysis (PCA) was performed with data from dry-hopped beers (the statistically significant sensory attributes from the Quantitative Descriptive Analyses and the content of key hop-derived volatile compounds) as active variables and liking scores as a passive supplementary variable (Figure 3).

The first two factors explained 93.15% of the variability of the data; the increased concentration of volatile compounds in the beers dry-hopped with PT11 and PT14 is notorious. There was a proximity in position of geraniol, ethyl 2- and 3-methylbutanoate, ethyl 4-methylpentanoate, S-methyl 5-methylpentanthioate and DMTS to the non-citrus fruit characteristic of PT11. There was also a closeness of ethyl 2-methylpropanoate, geranyl acetate, β-caryophyllene, 3MB2MP, 2MB2MP, S-methyl thiohexanoate, and ethyl cinnamate to citrus PT14 dry-hopped beer. Myrcene and linalool are equally distant from the citrus and non-citrus fruit attributes. The compound humulene oxide presented the largest squared cosine in the factor 3 axis (Supplementary Table S3); it was not possible to visualize (in the presented F1 vs. F2 graph) the proximity of this compound with the spicy note of PT05. Pearson correlation coefficients revealed strong correlations between the liking scores and non-citrus fruits attribute (r = 0.98). Regarding the volatile compounds, ethyl 2-methylpropanoate (r = 0.83), DMTS (r = 0.85), myrcene (r = 0.91), S-methyl 5-methylpentanthioate (r = 0.95) and linalool (r = 0.95) were the principal compounds related to the liking results. 

The sensory perception of beers was best explained by joining the sensory analysis and the quantification of hops-derived keys odour compounds. Therefore, the aroma composition of hops was evaluated using olfactometry, which takes into consideration both sensory and chemical qualities to provide a connection between hops and characteristics imprinted by dry-hopping.

### 3.4. Olfactometry of Hops vs. Sensory and Volatile Composition of Dry-Hoped Beers

Gas chromatography coupled with mass detection and olfactometry was applied to assess the odour-active compounds in 15 Portuguese wild hop samples (from which four were selected for dry-hopping beers). A total of 42 odour-active compounds were identified, including three aldehydes described as green fruity and grassy, three fruity, rancid, and cheese-like carboxylic acids, eight fruity (non-citrus) esters, two higher alcohols (green grassy and earthy), the ketone diacetyl (rancid), three monoterpenes (myrcene, limonene, and ocimene) and two monoterpenoids (linalool and geraniol) presenting resinous, spicy, citrus and floral perceptions, 12 thioesters producing sulphurous (garlic, onion, and cooked vegetable) but also earthy and fruity notes, and eight sulphurous thiols/ sulphides (Supplementary Table S2). The observed duality between the undesirable and pleasant description of sulphur compounds agrees with previous reports [17]. 

**Figure 4:**
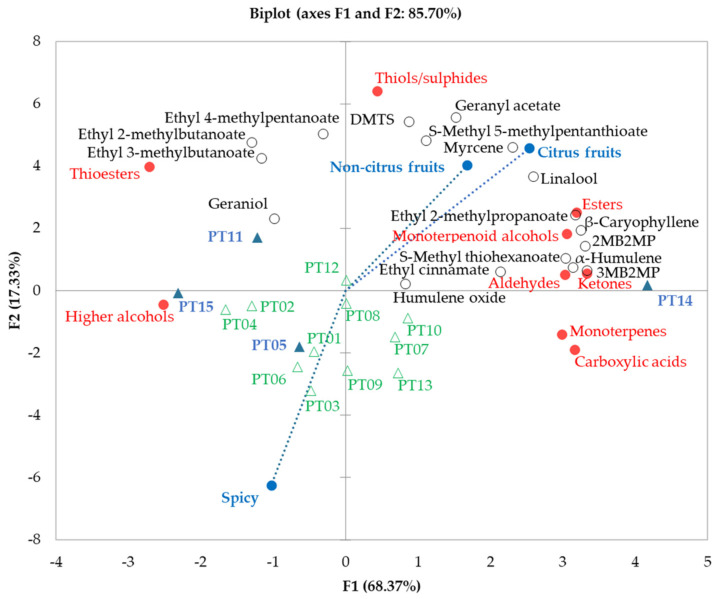
Principal Component Analysis (PCA) biplot using as active variables the olfactometry data (red circles) of the 4 hops (blue triangles), while the sensory attributes (blue circles) and volatile compounds quantified in the dry-hopped beers (open black circles) were used as passive (supplementary) variables and olfactometry data of the other 11 hop samples (open green triangles) were included as supplementary observations.

The sums of odour intensities for each class of compounds were calculated for each hops sample and were used as variables for the PCA, since the compounds from the same chemical functional group described above presented similar odour descriptions. Therefore, a PCA was performed to join the olfactometry data of hops with the sensory characteristics and volatile compounds content of dry-hopped beers. The active variables were the olfactometry data of the four hops used for beer production, while the sensory attributes and volatile compounds content in the dry-hopped beers were supplementary (passive) variables and the olfactometry data of the other 11 hop samples were supplementary observations (Figure 4). The first two component factors explained 85.70% of the total variance in the relationship between the hops and the sum of odour intensities of the chemical classes of the odour-active compounds. Supplementary variables and observations were not used to calculate the coordinates and are displayed as a layer over the plotted correlation. According to the square cosine values (Supplementary Table S3), PT05 and PT11 hops and thiols/sulphides are best explained by the F2 axis. The hop samples PT14 and PT15, and the other eight classes of odour-active compounds, are best described by the F1 axis. PT14 hops presented the highest odour-intensities of esters, monoterpenoids alcohols, aldehydes, ketones, monoterpenes and carboxylic acids. PT11 and PT15 hops showed more odour-intensity of thioesters, PT11 of thiols/sulphides and both, plus PT05, presented more odour-intensity of higher alcohols.

In addition, supplementary variables (sensory attributes and compounds quantified in beers) were used to understand how the chemical classes of hops’ odour-active compounds connected to the sensory and composition analysis of respective dry-hopped beers (Figure 4). Taking into consideration the Pearson correlation coefficients, it was possible to identify the relationship between the odour intensity of monoterpenoid alcohols and the citrus (r = 0.81) and non-citrus (r = 0.83) fruit sensations of beers, as well as with the concentration of linalool (r = 0.95). The odour intensity of the esters identified in olfactometry of hops presented a positive correlation (r = 0.94) with the citrus characteristic of dry-hopped beers, while ethyl 2-methylpropanoate (r = 0.98), 3MB2MP (r = 0.95), and 2MB2MP (r = 0.98) were the esters that presented strong correlations. These results are in total agreement with previous reports, reinforcing the connection between monoterpenoid alcohols and esters with citrus/fruity beer characteristics [11,17,22]. The odour intensity of thiol/sulphide compounds showed negative (r = −0.98) and positive correlations (r = 0.82) with spicy and non-citrus fruits, respectively. Data should be further investigated, since DMTS, the compound that showed an association (r = 0.98) with these results, is described as providing unpleasant sulphurous (garlic, onion, cooked vegetable) effects on beer flavour [34,35]. The odour intensities of thioesters, ketones, aldehydes, carboxylic acids, higher alcohols and monoterpenes in hops did not present any correlation with the sensory characteristics of dry-hopped beers.

Hops not used for brewing were employed as supplementary observations to find out how they are placed into the space of the odour-active compounds’ profiles of the four hops selected for brewing, and to hypothesize the composition and the spicy and fruits sensations of beers dry-hopped with these samples. These 11 hop samples seem to have more spiciness than fruitiness potential, because they are more related to PT05 and PT15 characteristics than to PT14.

## 4. Conclusions

Sensory analysis showed that Portuguese wild hops can satisfy the requirements of the modern brewing industry trends, differing in fruity (citrus and non-citrus), resinous, earthy, floral, green grassy, green tea, and sulphurous perceptions. Selected hop samples promoted fruity and spicy notes in dry-hopped beers, which presented increased contents of key hop-derived aroma compounds such as myrcene, geraniol, linalool, ethyl 2-methylpropanoate, 3MB2MP and 2MB2MP. Olfactometry coupled with PCA analysis of hop-derived volatile compounds revealed the connection between the odour intensity of monoterpenoid alcohols and esters, the fruitiness potential of hops, and beer composition. In addition, the Portuguese wild hops populations evaluated demonstrated their potential to impart mostly a spiciness aroma. 

This work presents an innovative approach to assessing data from the olfactometry of hops by using the sums of odour intensities from odour-active compounds of the same chemical functional group with similar odour descriptions. This was demonstrated as an efficient tool for complementing sensory and volatile compounds analyses and explaining the sensory perception that hops impart to dry-hopped beer, using reduced sampling for beer production. This approach can be applied to studies of new hops genotypes obtained either from wild populations surveys or breeding, at a fraction of the cost and time usually spent on these endeavours.

## Figures and Tables

**Table 1:** Attributes, standard and hop references provided to panellists during training sessions.

Attributes		Standard References
Fruity		
Citrus fruits	Lemon-, orange-, tangerine-like	Citral 30 µg/L
Not citrus fruits	Green fruits-like (pear, apple)	Hexanol 70 µg/L
	Red berries-like	Ethyl 3-methylbutanoate 40 µg/L
	Tropical fruits-like (pineapple, strawberry)	Ethyl hexanoate 20 µg/L
	Sweet fruit-like (banana, ice bonbon)	Isoamyl acetate 1.1 mg/L
**Vegetal**		
Resinous	Woody-like	Borneol 6 µg/L
	Pine-, cedar-like	(+) -β-Pinene 80 µg/L
Earthy	Mushroom-like	1-Octen-3-ol 100 µg/L
Floral	Floral-like	Linalool 7 µg/L
	Rose-like, floral-, honey-like	Phenyl ethanol 10 mg/L
Green	Grassy-like	*cis*-3-hexen-1-ol 0.25 µg/L
	Menthol (herbal)	Menthol 600 mg/L
	Tea-like	Theaspirane 4 µg/L
**Spice**		
Spicy	Curry-, cloves-like	Eugenol 130 µg/L
	Herbal, spicy	Myrcene 0.1 mg/L
**Others**		
	Cheese-like	Butyric acid 240 µg/L
	Cream caramel-like (sweet-like)	Furaneol 4 µg/L
	Cooked vegetable-like	Dimethyl sulphide 0.1 mg/L
	Butter-like (rancid)	Diacetyl 6.5 µg/L
	Sulphurous (garlic-, onion-, leek-like)	Dimethyl trisulphide 25 µg/L
	Sweaty-, cheese-like	Ethyl butanoate 240 µg/L
**Hop References**	**Attributes and Descriptions ^1^**	
Ariana	Fruity: black berries, blackcurrant, peach, pear, tropical fruits, resinous, grapefruit, strawberry, quince, green pepper, banana	
Bravo™	Citrus and herbal: orange, fruity, vanilla, floral, chrysanthemum, vanilla cream, vegetable, calendula, butter	
Hallertauer Blanc	Fruity and spicy: white wine, coffee, cassis, gooseberry, grapefruit, lemon grass, elderflower, grapes	
Nugget	Fruity, spicy, and resinous: pineapple, lemon, ginger, geranium, floral, lychee	
Hallertauer Magnum	Spicy and green fruits: fruity, apple, pepper, lemon, chocolate, green peppers, mint	
Hallertauer Taurus	Spicy and fruity: pepper, lime, currant, spicy, plain chocolate, ripe banana, pepper, curry	
Hallertauer Tradition	Herbal and citrus: tea, spicy, orange, lavender, cassis, apricot, citrus, peach	
Herkules	Citrus, fruity and spicy: pepper, spicy, resinous, orange, honeydew melon, lemon, melissa	
Hersbrücker	Herbal and green tea: spicy, hay, orange, tobacco, citrus, black tea, marjoram, ginger, melissa	
Hüll Melon	Fruity and sweet: melon, tropical fruit, orange, vanilla, fruit tea, wild strawberry, geranium, aniseed	
Mandarina Bavaria	Citrus and fruity: tangerine, grapefruit, lime, bubble gum, pineapple, gooseberry, cassis, strawberry, lemon	
Opal	Spicy: herbal, pepper, grass, aniseed, citrus, apricot, liquorice, aniseed, bergamot	
Polaris	Citrus and fruity: menthol, ice wine, pineapple, pineapple, woodruff, bergamot, banana, mint	
Smaragd	Herbal, spicy and resinous: spicy, aniseed, tobacco, clove, cognac, camomile tea, liquorice, tarragon, butter	

^1^ Adapted from Hopsteiner and Barth-Haas Group.

**Table 2:** Sensory analysis of Portuguese hops performed by Check-All-That-Apply (CATA) test.

Hops	Citrus Fruits	Non-Citrus Fruits	Resinous	Earthy	Floral	Green Grassy	Green Tea	Spicy	Sulphurous
PT01	35 ^a^	50 ^a,b^	35 ^a^	10 ^a,b^	30 ^a^	40	40	40	45 ^a,b^
PT02	30 ^a^	40 ^a,b^	70 ^a,b^	40 ^a,b^	20 ^a^	30	40	45	25 ^a,b^
PT03	20 ^a^	45 ^a,b^	**75** ^a,b^	15 ^a,b^	35 ^a,b^	10	45	5	25 ^a,b^
PT04	35 ^a^	60 ^a,b^	65 ^a,b^	15 ^a,b^	40 ^a,b^	30	45	55	05 ^a^
PT05	25 ^a^	40 ^a,b^	**85** ^b^	40 ^a,b^	25 ^a^	15	45	**80**	30 ^a,b^
PT06	30 ^a^	50 ^a,b^	**80** ^a,b^	20 ^a,b^	30 ^a^	5	60	70	15 ^a,b^
PT07	15 ^a^	10 ^a^	**85** ^b^	45 ^b^	20 ^a^	30	40	40	25 ^a,b^
PT08	40 ^a,b^	45 ^a,b^	70 ^a,b^	15 ^a,b^	30 ^a^	25	25	60	55 ^b^
PT09	20 ^a^	60 ^a,b^	**80** ^a,b^	25 ^a,b^	40 ^a,b^	20	5	55	15 ^a,b^
PT10	15 ^a^	55 ^a,b^	**75** ^a,b^	15 ^a,b^	40 ^a,b^	35	35	5	15 ^a,b^
PT11	40 ^a,b^	**75** ^b^	70 ^a,b^	20 ^a,b^	45 ^a,b^	30	35	**75**	15 ^a,b^
PT12	40 ^a,b^	60 ^a,b^	50 ^a,b^	10 ^a,b^	30 ^a^	45	35	5	45 ^a,b^
PT13	25 ^a^	50 ^a,b^	**75** ^a,b^	30 ^a,b^	40 ^a,b^	45	55	40	25 ^a,b^
PT14	**85** ^b^	**90** ^b^	50 ^a,b^	00 ^a^	**80** ^b^	40	25	70	25 ^a,b^
PT15	45 ^a,b^	65 ^b^	50 ^a,b^	05 ^a,b^	50 ^a,b^	20	15	60	45 ^a,b^
*p*-values	0.000	0.000	0.006	0.004	0.005	0.148	0.124	0.100	0.013

Values indicate the proportion (%) that the attribute was identified by the panellists. Values in bold highlight when 75% or more panellists identified the attributes in the hops. Cochran’s Q test was performed to determine whether the attributes differed as a function of hop samples. Different superscript letters in the same column represent significant differences for the descriptor by multiple pairwise comparisons using the critical difference (Sheskin) procedure.

**Table 3:** Concentrations and thresholds (µg/L) of volatile compounds in beers dry-hopped with Portuguese wild hops.

Compound (ms/RI); Threshold	NDH Beer	PT05 Beer	PT11 Beer	PT14 Beer	PT15 Beer
Esters					
Ethyl 2-methylpropanoate (116/173); 1.1–5000	0.19(0.15–0.23) ^a^	0.69 (0.59–0.79) ^b,c^	**1.30 (1.03–1.57)** ^c,d^	**2.34 (2.25–2.43)** ^d^	0.33 (0.32–0.34) ^a,b^
Ethyl 2-methylbutanoate (71/846); 1.1	0.022 (0.020–0.025) ^a^	0.17 (0.17–0.17) ^b,c^	0.36 (0.34 -0.38) ^c^	0.13 (0.11–0.15) ^a,b^	0.15 (0.13–0.16) ^a,bc^
Ethyl 3-methylbutanoate (88/851); 2–1300	0.08 (0.07–0.09) ^a^	0.89 (0.88–0.91) ^b,c^	**2.02 (1.98–2.06)** ^c^	0.53 (0.49–0.58) ^a,b,c^	0.53 (0.48–0.58) ^a,b^
Ethyl 4-methylpentanoate (88/964); 1–18	0.03 (0.02–0.03) ^a^	0.79 (0.76–0.81) ^a,b,c^	**1.96 (1.93–1.99)** ^c^	0.85 (0.80–0.90) ^b,c^	0.50 (0.46–0.55) ^a,b^
3-Methylbutyl 2-methylpropanoate (71/1008);> 30	0.22 (0.21–0.23) ^a^	0.72 (0.71–0.72) ^a,b^	1.50 (1.46–1.53) ^c,d^	**49.02 (41.75–56.28)** ^d^	0.92 (0.88–0.96) ^bc^
2-Methylbutyl 2-methylpropanoate (71/1012); 50–60	2.6 (2.5–2.6) ^a^	9.9 (9.8–10.0) ^a,b^	21.9 (21.7–22.0) ^c,d^	**89.0 (74.4–103.6)** ^d^	10.8 (10.2–11.3) ^b,c^
Ethyl cinnamate (176/1482); n.i.	0.10 (0.09–0.11) ^a^	0.88 (0.84–0.91) ^a,b^	0.99 (0.91–1.08) ^a,b,c^	3.79 (2.98–4.60) ^c^	2.79 (2.67–2.90) ^b,c^
**Monoterpenes**					
Myrcene (136/991); 9–1000	4.8 (4.6–5.1) ^a^	**126.1 (119.0–133.1)** ^a,b^	**235.8 (221.9–249.7)** ^b^	**249.7 (227.6–271.9)** ^b^	**110.5 (100.2–120.8)** ^a,b^
Geranyl acetate (93/1385); n.i.	0.07 (0.05–0.08)	0.83 (0.82–0.85)	1.71 (1.54–1.88)	1.89 (1.56–2.22)	1.52 (1.47–1.57)
**Monoterpenoid Alcohols**					
Linalool (80/1099); 1–100	**2.38 (2.37–2.39)** ^a^	**79.2 (78.9–79.5)** ^a,b^	**141.6 (135.2–148.0)** ^b^	**163.6 (134.0–193.3)** ^b^	**48.2 (46.9–49.5)** ^a,b^
Geraniol (69/1257); 4–500	0.120 (0.116–0.124) ^a^	**5.80 (5.37–6.23)** ^a,b^	**9.82 (9.32–10.32)** ^b^	1.22 (1.00–1.44) ^a,b^	0.15 (0.11–0.18) ^a^
**Sesquiterpenes**					
β-Caryophyllene (133/1442); 160–450	0.018 (0.017–0.018) ^a^	3.7 (3.4–4.0) ^a,b^	9.9 (9.7–10.1) ^c,d^	30.6 (25.7–35.4) ^d^	5.1 (4.8–5.4) ^b,c^
α-Humulene (80/1476); 50–630	0.28 (0.26–0.29) ^a^	1.48 (1.46–1.50) ^a,b^	3.58 (3.52–3.65) ^b,c^	**59.22 (48.92–69.52)** ^d^	12.36 (11.60–13.12) ^c,d^
**Sesquiterpenoid oxide**					
Humulene oxide (138/1633); 10–450	0.0004(0.0004–0.0005) ^a^	0.0130 (0.0127–0.0132) ^b,c^	0.0131 (0.0129–0.0134) ^c^	0.0104 (0.0104–0.0105) ^a,b,c^	0.0049 (0.0047–0.0050) ^a,b^
**Thiol/Sulphide**					
Dimethyl trisulphide (DMTS) (126/977); 0.000027–0.0001	**0.006 (0.006–0.007)** ^a^	**0.014 (0.011–0.016)** ^bc^	**0.038 (0.031–0.045)** ^d^	**0.025 (0.023–0.026)** ^cd^	**0.009 (0.009–0.010)** ^ab^
**Thioesters**					
S-Methyl 5-methylpentanthioate (131/1057); 15	0.00007 (0.00006–0.00007) ^a^	0.63 (0.61–0.65) ^b,c^	1.67 (1.63–1.72) ^d^	1.13 (0.95–1.30) ^c,d^	0.23 (0.22–0.24) ^a,b^
S-Methyl thiohexanoate (131/1093); 0.3–1.0	0.0016 (0.0006–0.0026) ^a^	**0.52 (0.49–0.55)** ^a,b^	**0.62 (0.58–0.66)** ^a,b,c^	**1.67 (1.49–1.85)** ^c^	**0.83 (0.59–1.06)** ^b,c^

Non-dry-hopped beer (NDH beer): multiple pairwise comparisons using the Conover-Iman procedure/Two-tailed test were performed for data without normal distribution, expressed as a median (minimum–maximum). Different superscript letters in a row show statistically significant differences between medians at *p*-values < 0.05. ms/RI = fragment mass and retention index used for the identification and quantification of compounds. The values in bold are above the thresholds, considering the minimal levels found in beer retrieved from the Hop Flavour Database http://methods.asbcnet.org/hop_Flavors_Database.aspx (accessed on 13 April 2021), n.i. = not informed.

## Supplementary Materials

The following are available online at https://www.mdpi.com/article/10.3390/foods10061397/s1. Table S1: Sum of significant attributes tables across assessor from sensory analysis of Portuguese hops performed by the Check-All-That-Apply (CATA) test. Table S2: Odour-active compounds of Portuguese wild hops. Identification and intensity determination by olfactometry (GC−O) with the method of aroma intensity. Table S3: Squared cosines of the main components (Factor 1 to 3) compounds (variables) and hops sample (observations), obtained by Principal Component Analysis (PCA) of GC-O results.
