# Supplementary material for: Sensory and Olfactometry Chemometrics as Valuable Tools for Assessing Hops’ Aroma Impact on Dry-Hopped Beers: A Study with Wild Portuguese Genotypes
Source: Foods. 2021 Jun 17;10(6):1397. doi: 10.3390/foods10061397 (PMC8234261; doi:10.3390/foods10061397)
Supplement: Supplementary file 1 [file foods-10-01397-s001.zip › foods-1237520-supplementary.pdf]

foods

Appendix A

Supplementary Table S1. Sum of significant attributes tables across assessor from sensory analysis of Portuguese hops performed by Check-All-That-Apply (CATA) test.

Hops	Citrus fruits	Non-citrus fruits	Resinous	Earthy	Floral	Sulphurous
PT01	7	10	7	2	6	9
PT02	6	8	14	8	4	5
PT03	4	9	15	3	7	5
PT04	7	12	13	3	8	1
PT05	5	8	17	8	5	6
PT06	6	10	16	4	6	3
PT07	3	2	17	9	4	5
PT08	8	9	14	3	6	11
PT09	4	12	16	5	8	3
PT10	3	11	15	3	8	3
PT11	8	15	14	4	9	3
PT12	8	12	10	2	6	9
PT13	5	10	15	6	8	5
PT14	17	18	10	0	16	5
PT15	9	13	10	1	10	9

Supplementary Table S2. Squared cosines of the main components (Factor 1 to 3) compounds (variables) and hops sample (observations), obtained by Principal Component Analysis (PCA) of GC-O results.

PCA of sensory attributes and compounds content of dry-hopped beers (Figure 3)	F1	F2	F3
Ethyl 2-methylpropanoate	**0.883**	0.064	0.053
Ethyl 2-methylbutanoate	0.092	**0.833**	0.075
Ethyl 3-methylbutanoate	0.094	**0.884**	0.022
Ethyl 4-methylpentanoate	0.306	**0.673**	0.021
Dimethyl trisulphide	**0.667**	0.320	0.012
Myrcene	**0.987**	0.013	0.000
3-Methylbutyl 2-methylpropanoate	**0.527**	0.429	0.045
2-Methylbutyl 2-methylpropanoate	**0.654**	0.318	0.028
S-Methyl 5-methylpentanthioate	**0.686**	0.313	0.001
S-Methyl thiohexanoate	0.423	**0.577**	0.000
Linalool	**0.963**	0.006	0.031
Geraniol	0.041	**0.927**	0.031
Geranyl acetate	**0.590**	0.059	0.351
β-Caryophyllene	**0.712**	0.276	0.013
Ethyl cinnamate	0.120	**0.813**	0.067
α-Humulene	0.433	**0.561**	0.006
Humulene oxide	0.167	0.404	**0.429**
Citrus fruits	**0.923**	0.043	0.034
Non-citrus fruits	**0.770**	0.196	0.033
Spicy	**0.749**	0.135	0.116
PT05	**0.708**	0.075	0.217
PT11	0.247	**0.732**	0.021
PT14	**0.612**	0.380	0.007
PT15	**0.684**	0.199	0.117
liking	**0.774**	0.096	0.130

The results corresponding to the supplementary variables and observations are displayed in the second part of the table. Values in bold correspond for each observation to the factor for which the squared cosine is the largest.

Supplementary Table S3. Odour-active compounds of Portuguese wild hops. Identification and intensity determination by olfactometry (GC–O) with the method of aroma intensity.

Ri[a]	Compound	Odour descriptors	PT01	PT02	PT03	PT04	PT05	PT06	PT07	PT08	PT09	PT10	PT11	PT12	PT13	PT14	PT15
Aldehydes																	
644	3-Methylbutanal[+]	Fruity, green fruit	0.0	0.0	0.0	0.0	0.0	0.0	0.0	0.0	0.0	0.0	0.0	0.0	1.0	1.0	0.5
652	2-Methylbutanal[+]	Fruity, green fruit	0.5	0.0	0.0	0.0	0.0	0.0	0.5	0.0	0.0	0.0	0.0	0.0	0.0	2.0	0.5
801	Hexanal[+]	Green grassy	0.0	0.0	0.0	0.0	0.0	0.0	0.0	0.0	0.0	0.0	0.0	0.0	0.0	1.0	0.0
Carboxylic acids																	
741	2-Methylpropanoic acid	Fruity, rancid, cheese	0.0	0.0	0.0	0.0	0.0	0.0	0.5	0.5	0.0	0.0	0.0	0.0	0.5	1.0	0.0
831	3-Methylbutanoic acid	Rancid, cheese	3.0	1.0	2.5	0.5	2.5	2.5	3.0	2.5	1.5	1.0	1.0	0.5	2.5	3.0	0.5
843	2-Methylbutanoic acid	Fruity, rancid, cheese	0.5	0.0	0.5	0.5	1.0	0.0	0.0	0.0	1.0	0.7	1.0	2.5	1.0	1.0	0.5
Esters																	
678	Methyl 2-methylpropanoate	Fruity, tropical fruit	0.0	0.0	0.0	0.0	0.0	0.5	0.0	0.5	0.5	0.0	0.0	0.0	0.5	1.0	0.0
753	Ethyl 2-methylpropanoate[+]	Fruity, sweet fruit	0.0	1.0	0.0	0.0	0.5	0.5	0.0	0.0	0.0	0.3	1.0	0.5	0.0	1.0	1.0
774	Methyl 2-methylbutanoate	Fruity, green fruit	2.0	1.0	1.0	1.0	1.5	1.0	1.0	2.0	1.0	0.3	1.5	1.0	1.5	1.5	1.0
848	Ethyl-2-methylbutanoate[+]	Fruity, tropical fruit	1.0	0.0	0.0	0.0	0.0	0.0	0.0	0.0	1.0	0.0	0.5	1.0	0.0	1.0	0.5
889	Methyl 4-methylpentanoate	Fruity, sweet fruit, tropical fruit	0.0	0.0	0.0	0.0	0.0	0.0	0.5	0.5	0.0	0.0	0.0	0.5	0.0	0.0	0.0
964	Ethyl 4-methylpentanoate[+]	Fruity, sweet fruit, tropical fruit	1.0	1.0	0.0	1.0	1.0	1.0	1.0	1.5	1.0	1.0	1.0	2.0	0.0	2.0	1.0
1062	Methyl 2-methylheptanoate	Fruity, tropical fruit	0.0	0.0	0.0	0.0	0.5	0.0	0.0	0.0	0.0	0.0	0.5	0.0	0.0	1.0	0.5
1182	Methyl phenylacetate	Honey, floral, sweet-like, fruity	0.5	0.5	0.0	0.0	0.5	0.0	0.0	1.0	0.0	0.3	1.0	0.0	0.0	1.0	0.0
Higher alcohols																	
868	3-Hexenol[+]	Green grassy	0.0	0.0	0.0	0.0	1.0	0.0	0.0	0.0	0.0	0.0	1.0	0.0	0.0	1.0	0.5
980	1-Octen-3-ol[+]	Mushroom, earthy	1.0	1.0	1.5	1.0	1.0	0.5	0.5	1.0	0.5	0.3	1.0	1.0	0.0	0.0	1.0
Ketone																	
545	Diacetyl[+]	Rancid	0.0	0.0	0.0	0.0	0.0	0.0	0.0	0.0	0.5	0.3	0.0	0.0	0.0	0.5	0.0
Monoterpenes																	
991	Myrcene[+]	Herbal, resinous, spicy	1.0	2.5	2.5	3.0	3.0	2.5	3.0	3.0	1.0	2.7	2.5	3.0	2.5	2.5	1.0
1036	Limonene[+]	Citrus, fruity	0.0	0.0	0.0	0.0	1.0	0.5	0.0	0.5	0.0	0.0	0.5	0.0	0.0	0.5	0.5
1050	Ocimene	Citrus, fruity, herbal	0.0	0.0	0.0	0.0	0.0	0.0	0.0	0.0	0.0	0.3	0.0	0.0	0.0	2.0	0.0
Monoterpenoid alcohols																	

1099	Linalool[+]	Floral, citrus, fruity	3.0	2.5	2.5	1.0	2.0	1.5	3.0	2.5	2.5	2.7	2.5	2.5	2.5	3.0	1.0
1257	Geraniol[+]	Floral, citrus, fruity	1.0	0.5	2.0	1.0	1.0	1.0	2.0	0.5	1.0	2.3	1.0	1.0	1.0	1.5	1.0
Thioesters																	
798	S-methyl propanthioate	Sulphurous	0.0	0.0	0.0	0.0	0.0	0.0	0.0	0.0	0.5	0.0	1.0	0.0	0.0	0.0	0.0
849	S-methyl-2-methylpropanthioate	Sulphurous	0.0	0.0	0.0	0.5	0.0	0.0	0.0	0.0	0.0	0.0	0.0	0.0	0.0	0.0	0.0
941	S-methyl 2-methylbutanthioate	Sulphurous, fruity Sweet, tropical, and green fruit	3.0	1.0	0.5	1.5	0.5	0.0	1.0	1.0	2.5	0.7	1.0	1.5	0.5	1.0	0.5
1059	S-methyl 4-methylpentanoate[+]	Sulphurous, garlic, onion, cooked vegetable	0.5	2.5	0.5	1.0	1.0	0.5	1.5	1.0	1.0	1.0	1.0	0.5	0.5	0.0	0.5
1093	S-methyl hexanthioate[+]	Sulphurous, garlic, onion, cooked vegetable	0.0	0.5	0.5	0.5	2.0	0.0	1.0	0.5	1.0	2.0	0.0	1.0	0.0	1.5	1.0
1168	S-methyl 5-methylhexanthioate	Sulphurous, garlic, onion, cooked vegetable	0.0	0.0	0.5	0.0	1.0	0.5	0.0	0.0	0.0	0.7	0.5	0.0	0.5	1.0	1.0
1200	S-methyl heptanthioate	Sulphurous, fruity, garlic, onion, cooked vegetable	0.0	0.0	0.0	0.0	0.0	0.0	0.0	0.0	0.0	0.0	0.0	0.0	0.5	0.0	1.0
1213	S-methylthiomethyl 2-methylpropanthioate	Sulphuro*us, garlic, onion	2.5	1.5	0.0	2.0	0.5	2.5	1.5	2.5	1.0	0.3	2.0	2.0	0.5	0.5	1.0
1307	S-methylthiomethyl 3-methylbutanthioate	Sulphurous, garlic, onion, earthy	0.0	0.0	0.0	0.5	0.0	0.0	0.0	0.0	0.0	0.0	1.0	0.0	0.0	0.0	1.0
1309	S-methylthiomethyl 2-methylbutanthioate	Sulphurous, garlic, onion, earthy	2.0	2.0	0.0	0.5	1.0	1.0	1.5	1.5	1.0	1.0	2.0	2.0	0.5	1.0	3.0
1381	S-methyl-phenylacet thioate	Sulphurous, solvent	0.0	0.0	0.0	0.0	0.0	0.5	0.0	0.0	0.0	0.0	0.0	0.0	0.0	0.0	0.0
1431	S-methylthiomethyl 4-methylpentanthioate	Sulphurous, garlic, onion, earthy	0.0	1.0	0.0	0.5	0.0	2.0	0.0	1.0	1.0	0.0	3.0	0.5	0.5	0.0	2.5
Thiols/ sulphides																	
400	Methyl mercaptan	Sulphurous	0.0	0.0	1.0	1.0	0.5	0.0	0.5	1.0	0.5	1.0	2.0	1.0	0.5	1.5	0.5
497	Dimethyl sulphide	Sulphurous	0.0	0.0	0.0	0.0	0.0	0.5	0.0	0.0	0.0	0.0	0.0	0.0	0.0	0.0	0.0
747	Dimethyl disulphide[+]	Sulphurous, garlic, onion	0.0	0.0	0.0	0.5	0.5	0.0	0.0	0.0	0.0	0.0	0.0	0.0	0.0	1.0	0.0
808	C2H4S2	Sulphurous	2.0	2.5	2.5	3.0	2.5	2.0	2.0	3.0	1.0	2.3	2.5	3.0	2.5	3.0	3.0
977	Dimethyl trisulphide[+]	Sulphurous, garlic, onion	0.5	1.5	0.5	1.0	0.5	1.0	0.5	0.5	1.0	2.3	0.5	1.0	0.0	0.0	0.5
1135	Trithio-2,3,5-hexane	Sulphurous	2.0	3.0	1.0	2.5	1.0	1.5	2.5	2.5	1.0	1.0	3.0	3.0	2.0	1.5	2.0
1538	Tetrathio-2,4,5,7-octane	Sulphurous, garlic, onion, earthy	0.0	0.0	0.0	0.0	0.5	0.0	0.5	0.5	0.0	0.0	1.0	0.5	0.5	0.5	1.0
1272	3-(4-Methyl-3-pentenyl)-thiophene	Sulphurous	0.0	0.0	0.0	0.0	1.0	0.0	0.0	0.0	0.0	0.0	0.5	0.0	0.0	0.5	0.0

[a] calculated by linear interpolation from the retention times of the compound and adjacent n-alkanes. Compounds were identified by retention index (RI), odour descriptors, and confirmed by authentic standards ($^{+}$) and mass spectrum library. Values represent the average of analysis of two or three sniff sections.
